# Brusatol enhances MEF2A expression to inhibit RCC progression through the Wnt signalling pathway in renal cell carcinoma

**DOI:** 10.1111/jcmm.17972

**Published:** 2023-10-20

**Authors:** Tao Wang, Yu Zhou, Hui Bao, Bo Liu, Min Wang, Lei Wang, Tiejun Pan

**Affiliations:** ^1^ Department of Urology General Hospital of the Central Theater Command Wuhan China; ^2^ Department of Urology Renmin Hospital of Wuhan University Wuhan China

**Keywords:** Brusatol, MEF2A, renal cell carcinoma, ubiquitylation, Wnt/beta‐catenin

## Abstract

Renal cell carcinoma (RCC) is the most aggressive subtype of kidney tumour with a poor prognosis and an increasing incidence rate worldwide. Brusatol, an essential active ingredient derived from Brucea javanica, exhibits potent antitumour properties. Our study aims to explore a novel treatment strategy for RCC patients. We predicted 37 molecular targets of brusatol based on the structure of brusatol, and MEF2A (Myocyte Enhancer Factor 2A) was selected as our object through bioinformatic analyses. We employed various experimental techniques, including RT‐PCR, western blot, CCK8, colony formation, immunofluorescence, wound healing, flow cytometry, Transwell assays and xenograft mouse models, to investigate the impact of MEF2A on RCC. MEF2A expression was found to be reduced in patients with RCC, indicating a close correlation with MEF2A deubiquitylation. Additionally, the protective effects of brusatol on MEF2A were observed. The overexpression of MEF2A inhibits RCC cell proliferation, invasion and migration. In xenograft mice, MEF2A overexpression in RCC cells led to reduced tumour size compared to the control group. The underlying mechanism involves the inhibition of RCC cell proliferation, invasion, migration and epithelial‐mesenchymal transition (EMT) through the modulation of Wnt/β‐catenin signalling. Altogether, we found that MEF2A overexpression inhibits RCC progression by Wnt/β‐catenin signalling, providing novel insight into diagnosis, treatment and prognosis for RCC patients.

## 1 INTRODUCTION

Renal cell carcinoma (RCC) incidence is significantly increasing worldwide, especially for youngsters.^1^ Clear cell renal cell carcinoma (ccRCC) is the predominant subtype of renal cell carcinoma, characterized by significant cancer heterogeneity, distinct clinical progression and potential therapeutic vulnerabilities.^2^ Thus, exploring effective drugs and possible molecular mechanisms for diagnosis, treatment and prognosis in RCC patients is particularly essential.

Brusatol, a particularly bioactive composition of Brucea javanica, possessed the antitumor function in multiple tumours,^3^ including melanoma,^4^ head and neck squamous cell carcinoma,^5^ pituitary adenoma,^6^ nasopharyngeal carcinoma^7^ and breast cancer.^8^ Our previous study found that brusatol disturbing the RCC progression by PTEN/PI3K/AKT pathway,^9^ and we believe brusatol‐associated studies will bring treatment breakthroughs for RCC patients. Thus, exploring brusatol could provide novel insight into cancer treatment, especially RCC.

The Wnt/β‐catenin signalling pathway plays a pivotal role in regulating essential cellular processes, including cell proliferation, apoptosis, stem cell self‐renewal, tissue homeostasis and wound healing. Dysregulation of this pathway is closely linked to tumorigenesis.^10^ Many studies found that the Wnt/β‐catenin pathway is essential in cancer. In the study of Zhang W et al., the Wnt/β‐catenin pathway is associated with thyroid cancer progression and crucial to maintaining stemness.^11^ Activation of the Wnt/β‐catenin signalling pathway expedites the progression of breast cancer.^12^ In hepatocellular carcinoma (HCC), the positive regulator of the Wnt/β‐catenin signalling pathway is the nucleoside diphosphate kinase 7 (NME7), which promotes HCC progression.^13^ The degradation of β‐catenin in the Wnt/β‐catenin signalling pathway is closely linked to ubiquitylation.^14^

Ubiquitylation is a biological process that plays crucial roles in cell communication, protein degradation, DNA repair and cell division.^15^ As we know, protein degradation, accounting for ubiquitylation, is led to the downregulation of protein expression. Downregulation of RNF128 enhances melanoma cell epithelial‐mesenchymal transition (EMT) and stemness by ubiquitinating and degrading CD44/CTTN.^16^ In the study of Li Y et al., the downregulation of MED1 promotes SMAD3 ubiquitinating, protects against SMAD3 degradation and decreases melanoma cell metastasis.^17^

In this study, we predicted 37 molecular targets of brusatol based on the structure of brusatol. Then, we identified a key target MEF2A as our object through bioinformatics analysis. Hence, brusatol and its target MEF2A hold promise as a novel therapeutic approach for inhibiting RCC progression.

## 2 MATERIALS AND METHODS

### 2.1 Data collection

Expression profiles and clinical information of renal cell carcinoma (RCC) patients were extracted from The Cancer Genome Atlas (TCGA) (https://portal.gdc.cancer.gov/). The dataset consisted of 72 normal samples and 541 tumour samples.

### 2.2 Human tissues

After obtaining patient consent, RCC tissues were acquired via surgical resection. All RCC samples were collected at Renmin Hospital of Wuhan University and immediately placed in a −80°C freezer. The ethics committee of Renmin Hospital of Wuhan University approved the collection and handling of human tissues.

### 2.3 Survival analysis and GSEA using the TCGA database

We utilized the R language to perform clinical correlation, survival and independent prognostic analyses, while GSEA software was employed to conduct the GSEA analysis. For clinical correlation analysis, we applied the ‘ggpubr’ R package. The ‘survival’ R package was used to perform survival analysis.

Gene Set Enrichment Analysis (GSEA) is a computational method used to evaluate whether a predefined set of genes exhibits statistically significant and consistent differences between two biological states or phenotypes.^18^, ^19^

### 2.4 Cell cultivation and transfection

Cell lines (HK‐2, 786‐O and 769‐P) were acquired from the cell bank of the Chinese Academy of Sciences (Shanghai, China). The cells were cultured in DMEM cell culture medium (Gibco, United States) supplemented with 10% FBS (Gemini, United States) and incubated at 37°C with 5% CO2.

The lentiviral vector (Lv‐MEF2A) for overexpressing MEF2A was obtained from RiboBio Company (Guangzhou, China) along with the control lentiviral plasmid (vector). RCC cells were seeded in six‐well plates and transduced with lentiviruses in the presence of polybrene (8 μg/mL). Following a 24‐h infection period, the culture medium was replaced with fresh medium supplemented with 3 μg/mL puromycin to selectively maintain successfully transduced cell lines, while eliminating uninfected RCC cells. The selected cell lines were used for further experiments.

### 2.5 RT‐PCR

Total RNA was extracted from RCC cells using RNA‐easy Isolation Reagent. The extracted RNA was reverse‐transcribed using the Thermo Fisher Scientific Reagent Kit, and cDNA was obtained. Subsequently, we conducted qRT‐PCR experiments using the cDNA, with primers listed in Table 1. The mRNA expression of MEF2A was calculated using 2−^△△ct^.

**TABLE 1:** Primers of RT‐PCR.

Primer name	Sequence(5′‐3′)
MEF2A (Forward)	AGCAGCCCTCAGCTCTCTTG
MEF2A (Reverse)	GGTGAAATCGGTTCGGACTTG
GAPDH (Forward)	GAACGGGAAGCTCACTGG
GAPDH (Reverse)	GCCTGCTTCACCACCTTCT

### 2.6 Western blot

We used a radioimmunoprecipitation assay buffer to obtain protein lysates from cells and tissues. Following that, protein lysates were subjected to 10% sodium dodecyl sulphate‐polyacrylamide gel electrophoresis (SDS‐PAGE) for protein separation, and subsequently transferred onto polyvinylidene fluoride (PVDF) membranes. The PVDF membranes were then blocked using 5% skimmed milk and incubated with primary antibodies at a dilution of 1:1000 (Abclonal provided antibodies against Vimentin, c‐myc, and Caspase‐3. Ki‐67 antibody was obtained from Abcam. Proteintech supplied the following antibodies: MEF2A, Bax, MMP‐9, Bcl‐2, E‐cadherin, N‐cadherin, Survivin, H3, β‐Actin, CyclinD1 and GAPDH). After thorough washing, the membranes were further incubated with horseradish peroxidase (HRP)‐conjugated secondary antibodies. Finally, western blotting was visualized using an imaging system.

### 2.7 CCK‐8 assay

Transfected RCC cells were plated at a density of 2000 cells per well in 96‐well plates. To assess cell proliferation, 10 μL of CCK‐8 solution (Boster, Pleasanton) was added to each well, and the plates were transferred to an incubator for 2 h. The absorbance at 450 nm was measured using a microplate reader.

### 2.8 Colony formation assay

Following successful transfection, 500 cells were seeded in each well of six‐well plates. After a 14‐day incubation period, the cells were fixed with 4% paraformaldehyde (PFA), stained with gentian violet and subsequently washed with phosphate‐buffered saline (PBS). The number of discernible colonies was then quantified as a measure of colony formation capacity.

### 2.9 Flow cytometry

To assess alterations in the cell cycle, cells were transfected and cultured until they reached 80% confluency. Then, they were washed with PBS and fixed in 70% ethanol overnight. After another day, cells were labelled with a fluorescent dye and analysed by PI flow cytometry to determine changes in the cell cycle.

To assess alterations in the cell apoptosis, cells were transfected and cultured to detect apoptosis until reaching 85% density. After fluorescence labelling, the integrity of the cell membrane was assessed by combining phospholipid‐binding protein and phosphatidylserine.

### 2.10 Transwell assay

Following successful transfection, 24‐well Transwell migration chambers (BD Biosciences, USA) were employed to assess the migratory and invasive properties of the cells. The chamber inserts were pre‐coated with a 1:8 ratio of Matrigel to DMEM. RCC cells were seeded in the upper chamber with serum‐free medium, while the lower chamber was filled with 800 μL of DMEM supplemented with 20% FBS. After a 24‐h incubation period, the cells were washed with PBS, fixed with 4% paraformaldehyde (PFA) and stained with gentian violet. Finally, cell layers were photographed using an Olympus BX51 microscope.

### 2.11 Wound healing assay

RCC cells were plated in a six‐well plate and cultured until they reached approximately 85% confluence. A 200 μL pipette tip was used to create a scratch on the cell monolayer. Subsequently, the cells were washed three times with serum‐free medium and cultured in a medium containing 2% FBS. Images of the scratch were captured using a microscope.

### 2.12 Immunofluorescence

After successful transfection, RCC cells were evenly seeded on slides. The cells were fixed with 4% paraformaldehyde (PFA) and blocked with goat serum. Then, the cells were incubated with primary antibody for over 24 h at 4°C. Following this, a fluorescently labelled secondary antibody was incubated for 70 min. Finally, nuclear staining with DAPI was performed.

### 2.13 Xenografts in mice

A total of six 4‐week‐old mice were procured from Hunan Slike Jingda Laboratory Animals (Changsha, China) to establish the xenograft model for the study. The mice were randomly assigned to two groups, namely pc‐Vector and Lv‐MEF2A. They were housed in a specific pathogen‐free (SPF) environment at the experimental animal base of Renmin Hospital, Wuhan University. RCC cells transfected with vector and Lv‐MEF2A were injected into the mice. Tumour growth was monitored at 5‐day intervals, and after 1 month, the mice were humanely euthanized. The tumour tissues were dissected, documented photographically and then stored either in liquid nitrogen or fixed in formaldehyde for further analysis.

### 2.14 Statistical analyses

Statistical analysis was conducted using R language, SPSS 22.0, and GraphPad Prism 8 software. Data analysis involved Student's *t*‐test and chi‐square tests as appropriate. The results are expressed as mean ± standard deviation (SD), unless stated otherwise. All experiments were repeated independently at least three times to ensure reproducibility. Statistical significance was defined as *p* < 0.05, denoted as *** for *p* < 0.001, ** for *p* < 0.01 and * for *p* < 0.05.

## 3 RESULTS

### 3.1 Identification of brusatol‐associated molecular targets and MEF2A

**FIGURE 1:**
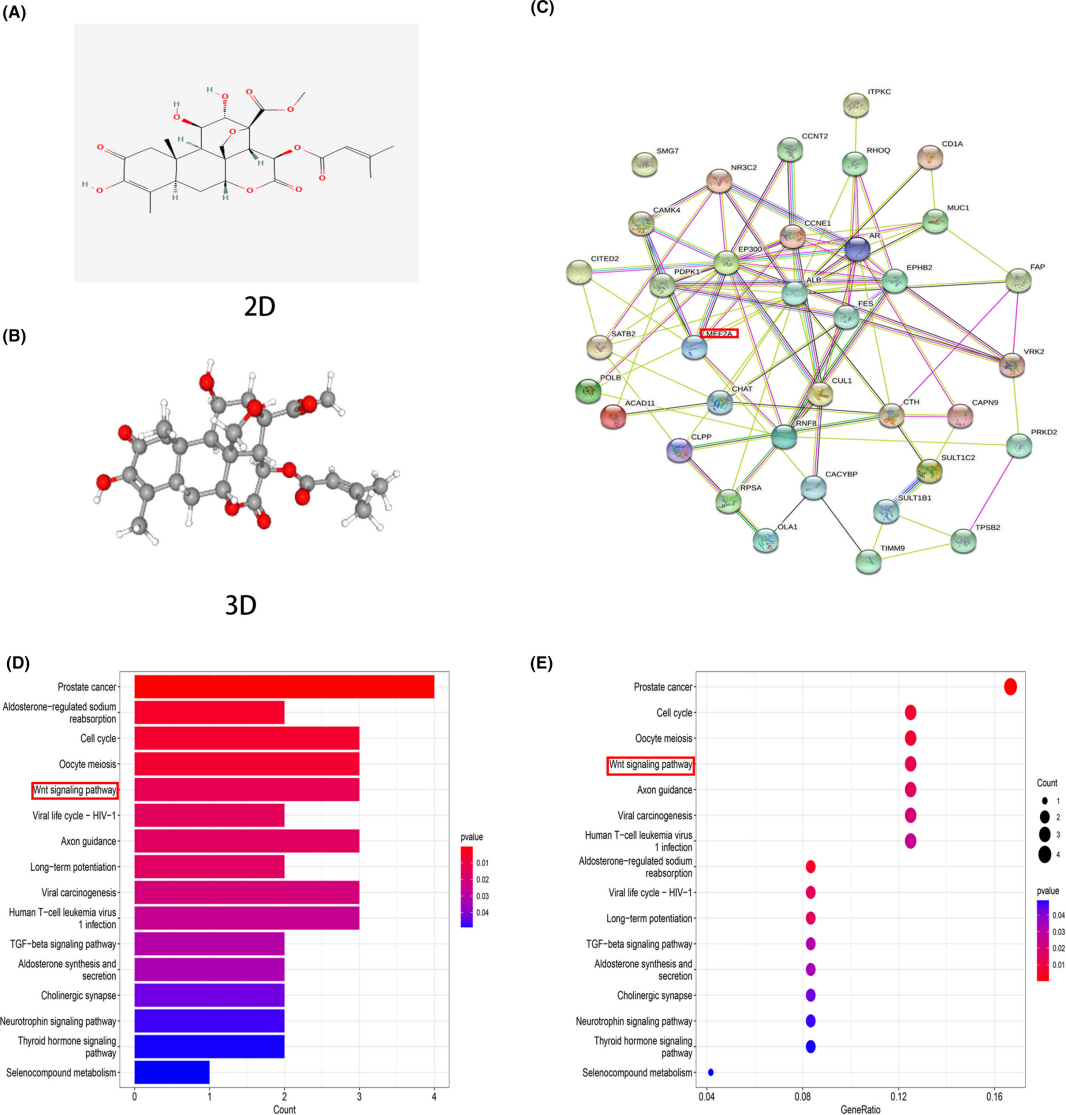
The molecular structures, molecular targets and potential mechanism analysis about brusatol. (A) 2D structure of brusatol based on online database. (B) 3D structure of brusatol based on online database. (C) 37 molecular targets of brusatol were predicted by String online tool. (D) Bar graph of KEGG to explore potential mechanism about brusatol. (E) Bubble graph of KEGG to explore potential mechanism about brusatol.

**FIGURE 2:**
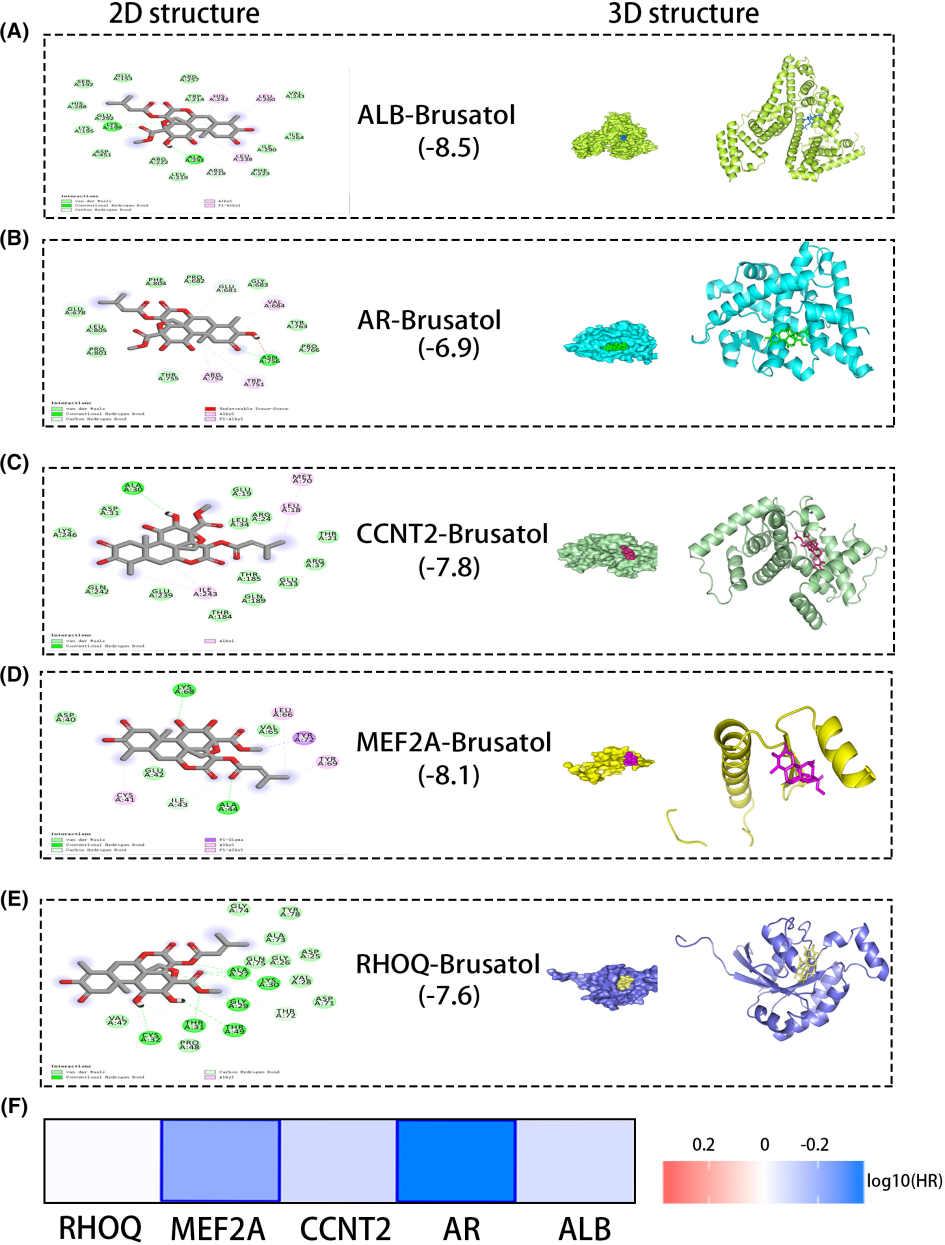
Through compared binding energies, MEF2A was selected as our objective. (A) The binding energy of ALB and brusatol. (B) The binding energy of AR and brusatol. (C) The binding energy of CCNT2 and brusatol. (D) The binding energy of MEF2A and brusatol. (E) The binding energy of RHOQ and brusatol. (F) Survival heatmap shows the correlation between key genes and overall survival (OS).

The two‐dimension (2D, Figure 1A) and three‐dimension (3D, Figure 1B) structures of brusatol were obtained from the online website (https://pubchem.ncbi.nlm.nih.gov/). The molecular targets of brusatol were predicted using an online tool (http://www.lilab‐ecust.cn/pharmmapper/) which identified 37 targets. The online String tool was used to visualize these targets and construct PPI (protein–protein interaction) network (Figure 1C). To explore the molecular mechanism, we performed KEGG analysis, which revealed that the molecular targets associated with brusatol were closely linked with the Wnt pathway, TGF‐beta pathway, etc. (Figure 1D,E). Cytoscape software was used to identify the key targets, and five key targets (ALB, AR, CCNT2, MEF2A and RHOQ) were identified using the 12 calculation methods, which included MCC (Figure S1A), Betweenness (Figure SB1), BottleNeck (Figure S1C), Closeness (Figure S1D), ClusteringCoefficient (Figure S1E), Degree (Figure S1F), DMNC (Figure S1G), EcCentricity (Figure S1H), EPC (Figure S1I), MNC (Figure S1J), Radiality (Figure S1K) and Stress (Figure S1L). Dock software generated molecular docking graphs and calculated the binding energy (Figures 2A–E). Through the survival heatmap, we discovered that only MEF2A and AR were closely associated with the overall survival of RCC patients (Figure 2F). Therefore, by comparison, MEF2A was selected as our target.

### 3.2 MEF2A deubiquitination led to low expression in RCC tissue

**FIGURE 3:**
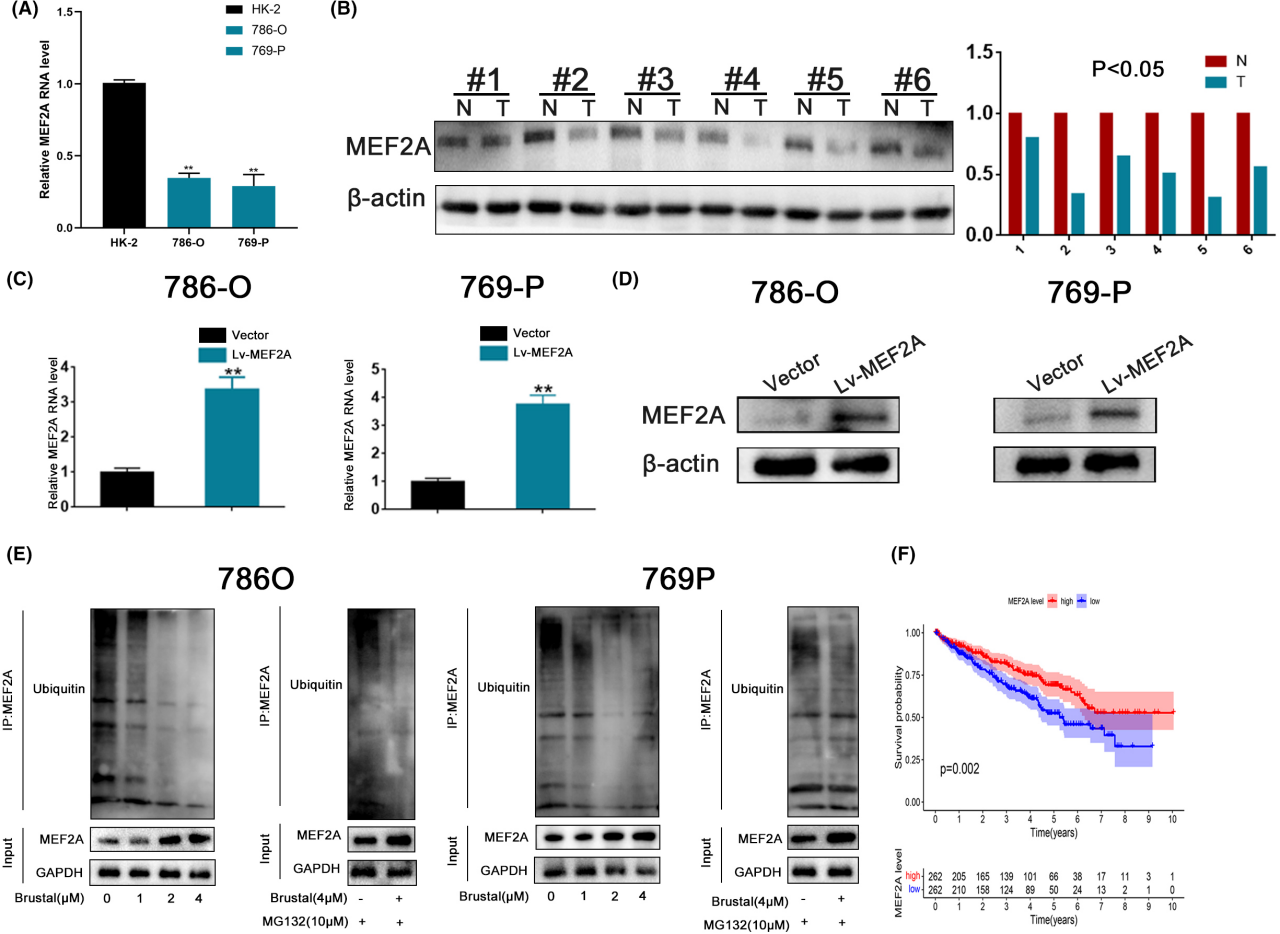
The expression of MEF2A, MEF2A expression mechanism and survival analysis. (A) RT‐PCR verified MEF2A expression in cell lines. (B) Western blot assay verified MEF2A expression in human tissues. (C) RT‐PCR verified MEF2A overexpression in cell lines. (D) Western blot assay verified MEF2A overexpression in cell lines. (E) The expression of MEF2A protein in renal cell carcinoma (RCC) cells transfected with indicated plasmids and treated with CHX for indicated time. (F) Brusatol inhibits MEF2A ubiquitination in cell lines. The downregulation of MEF2A is associated with shorter overall survival (OS) in TCGA database.

Firstly, we utilized an RT‐PCR assay to verify MEF2A expression levels in the normal cell (HK‐2) and RCC cells (786‐O and 769‐P). The result demonstrated that MEF2A expression is higher in the normal renal cell line compared to the RCC cell lines (Figure 3A). Moreover, western blot assay verified that MEF2A expression is lower in RCC carcinoma than in para‐carcinoma tissues (Figure 3B). So, we deemed that MEF2A expression is lower in RCC tumour tissues. Furthermore, we explored the reason for the low expression of MEF2A in RCC tissues. As we know, ubiquitination may lead to protein degradation, and we speculated that the low expression level of MEF2A is related to the MEF2A ubiquitination level. We constructed MEF2A overexpression lentiviral and used RT‐PCR (Figure 3C) and western blot (Figure 3D) to verify the transfection effect. To further observe hypothesis, we detected measured the half‐life of MEF2A protein in RCC cells treated with CHX. Results shows that brusatol inhibited the degradation of MEF2A through ubiquitination (Figure S2A,B). We performed a western blot ubiquitination assay in RCC cell lines (Figure S2C). The result observed that the brusatol inhibited MEF2A ubiquitination. Finally, through survival analysis, we discovered that MEF2A low expression is related to shorter overall survival (Figure 3F). Thus, we extrapolated that MEF2A deubiquitination could cause MEF2A expression upregulation and inhibits RCC progression.

### 3.3 MEF2A overexpression inhibits RCC cell proliferation

**FIGURE 4:**
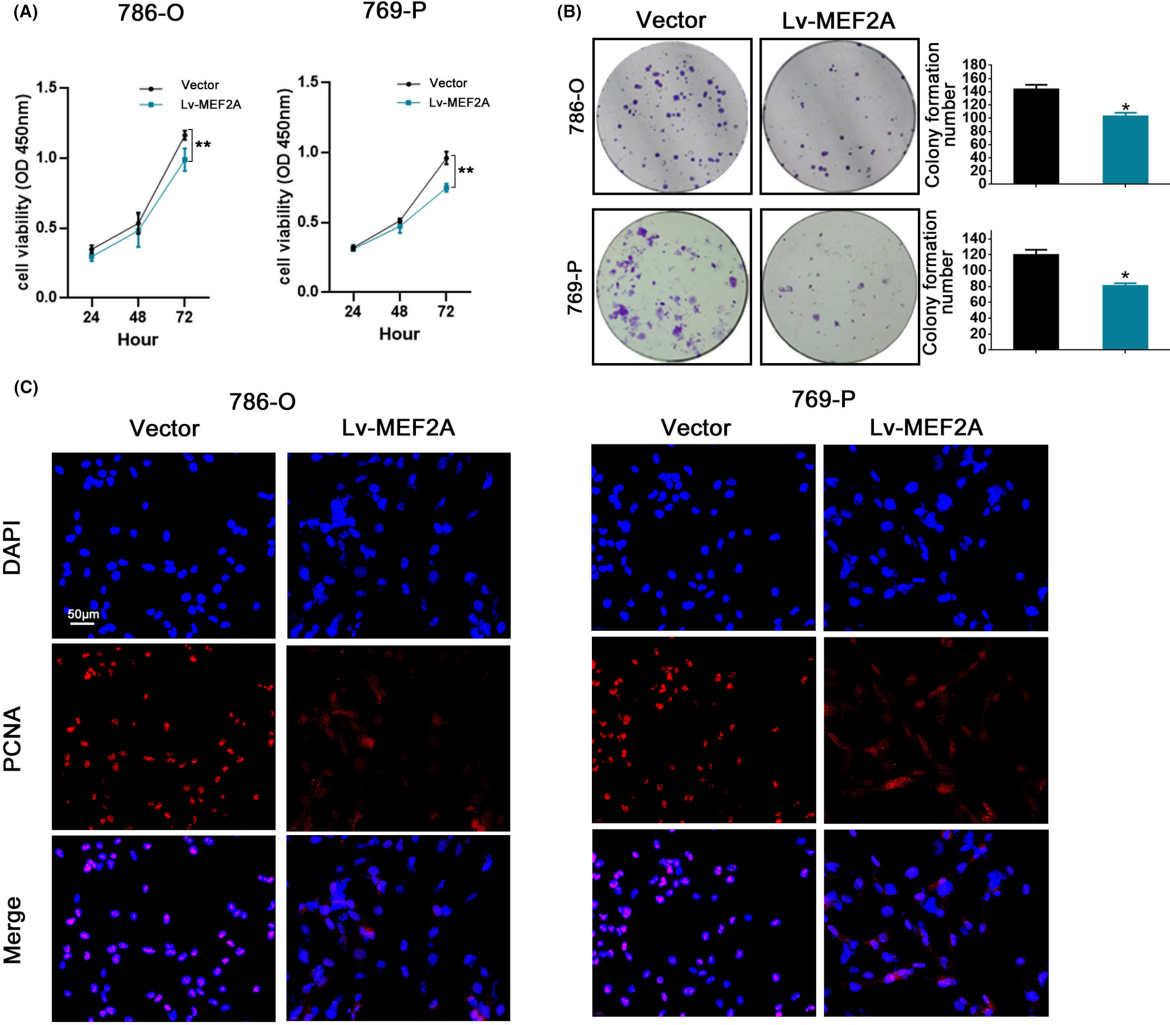
MEF2A inhibits renal cell carcinoma (RCC) progression in cell lines. (A) CCK‐8 proliferation assay after MEF2A overexpression. (B) Colony formation assay after MEF2A overexpression. (C) PCNA immunofluorescence assay after MEF2A overexpression.

**FIGURE 5:**
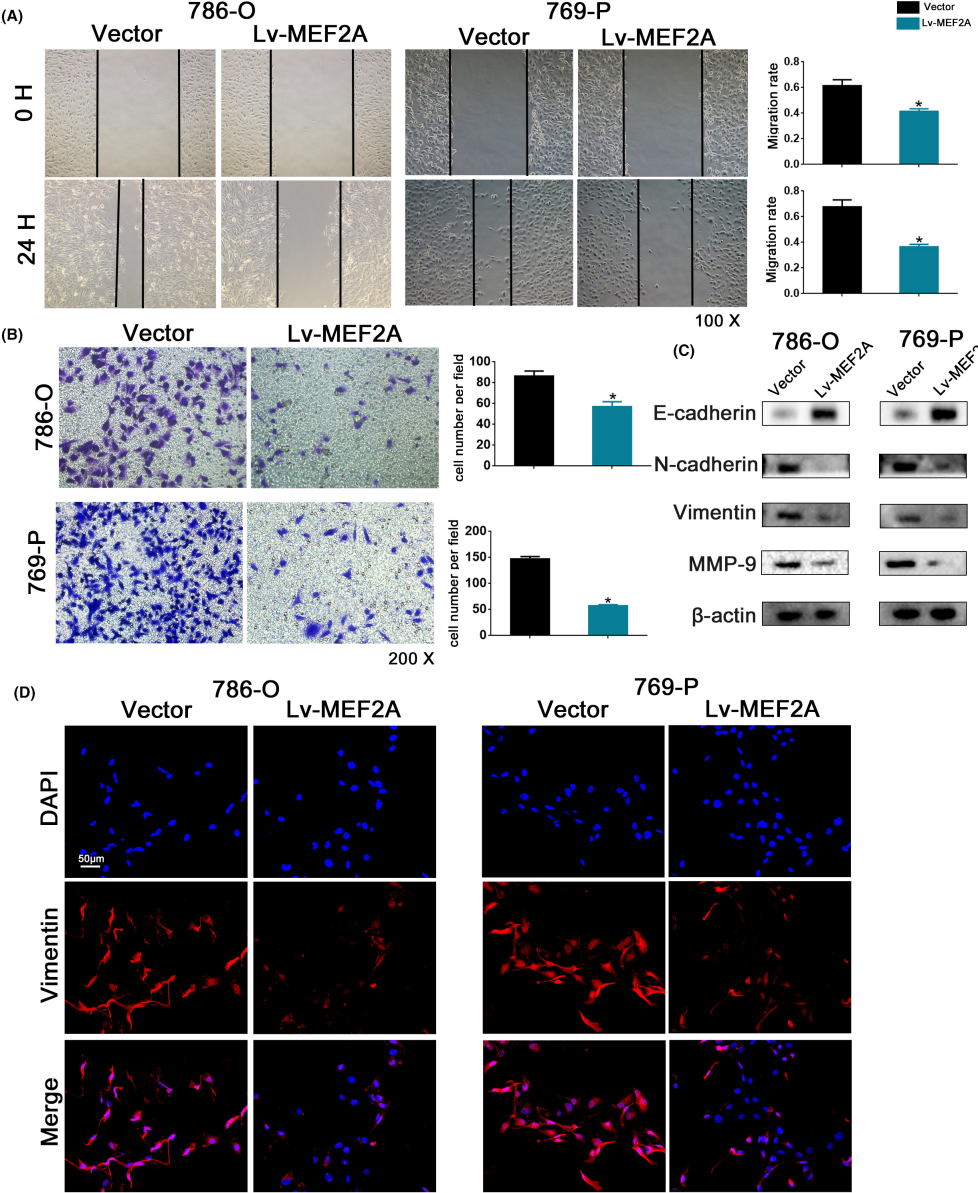
MEF2A inhibits renal cell carcinoma (RCC) progression in cell lines. (A) Wound healing assay after MEF2A overexpression. (B) Transwell migration assay after MEF2A overexpression. (C) Western blot assays verified EMT‐associated and proliferation‐associated protein expression. (D) Vimentin immunofluorescence assay after MEF2A overexpression.

To investigate the potential mechanism of MEF2A in RCC progression, we constructed a MEF2A overexpression group (Lv‐MEF2A). We performed CCK‐8 (Figure 4A) and colony formation (Figure 4B) assays to evaluate cell proliferation ability, which showed that proliferation and colony formation abilities were lower in the Lv‐MEF2A compared to the vector group. PCNA is a critical protein for DNA replication and tumour proliferation.^20^ Immunofluorescence analysis of PCNA (Figure 4C) revealed lower expression levels in the Lv‐MEF2A compared to the vector group. The wound healing assay revealed the Lv‐MEF2A group had reduced invasion ability compared to the vector group in RCC cells (Figure 5A). Moreover, Transwell assay justified the Lv‐MEF2A group had fewer migrating cells than the vector group in RCC cells (Figure 5B). E‐cadherin, N‐cadherin and Vimentin are essential proteins involved in EMT, while MMP‐9 is a critical protein in tumour proliferation. Western blot analysis revealed that MEF2A overexpression resulted in increased levels of E‐cadherin and decreased expression of N‐cadherin, Vimentin and MMP‐9 (Figure 5C). Immunofluorescence analysis of Vimentin also revealed reduced expression levels in the Lv‐MEF2A group (Figure 5D).

Therefore, we conclude that overexpression of MEF2A can suppress RCC cell proliferation, invasion, migration and EMT.

### 3.4 MEF2A overexpression inhibits RCC progression by influencing apoptosis and arresting the G1/S phase of the cell cycle

**FIGURE 6:**
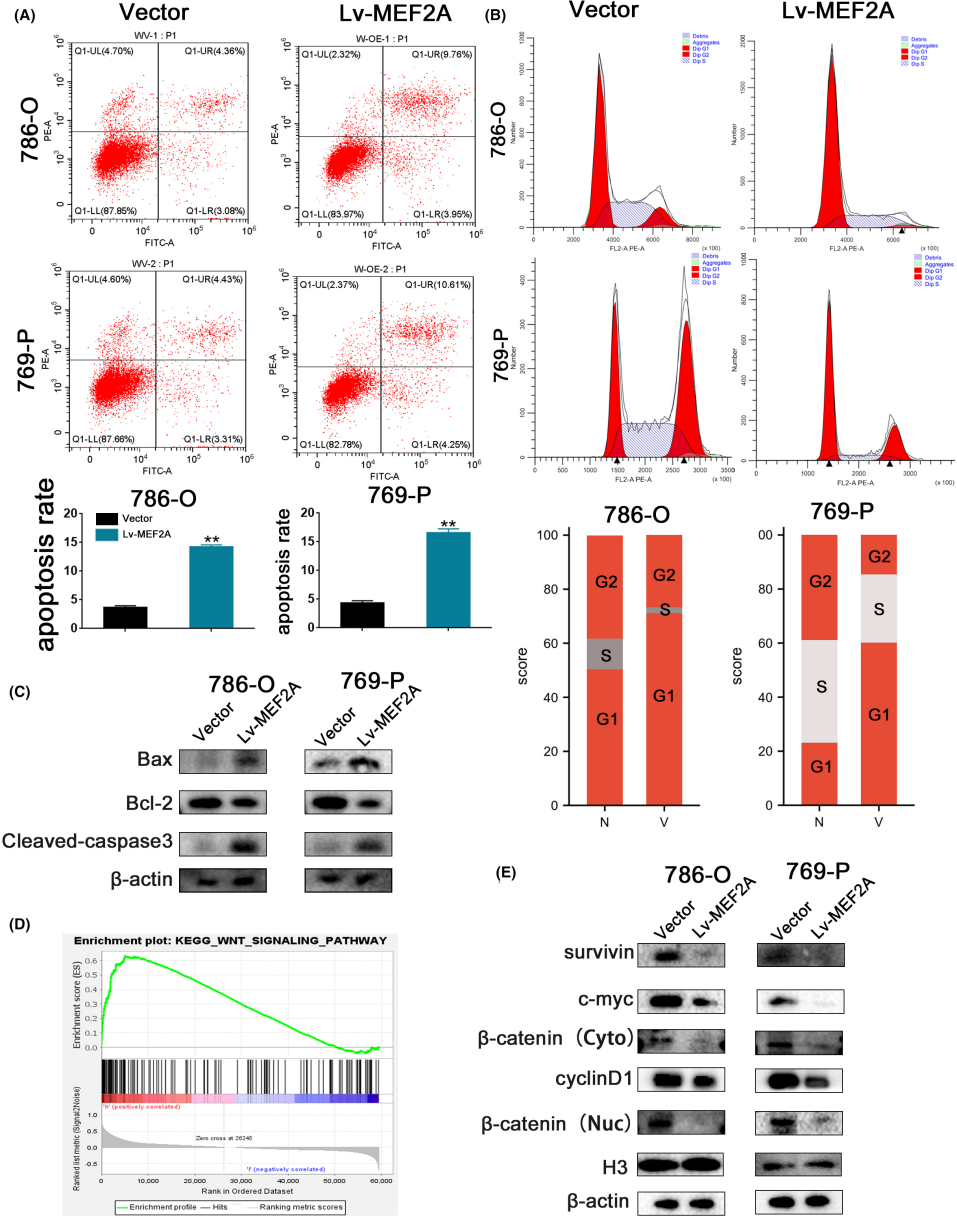
MEF2A overexpression promotes renal cell carcinoma (RCC) cell apoptosis, suppresses the G1/S transition of the cell cycle and inhibits the Wnt signalling pathway. (A) Cell apoptosis assay verified cell apoptosis ratio. (B) Cell cycle assay detected cell number of each phase of cell cycle. (C) Western blot assay tested apoptosis‐associated protein expression. (D) GSEA revealed that MEF2A influenced RCC progression by the Wnt signalling pathway. (E) Western blot assay verified Wnt‐associated protein expression.

Flow cytometry was applied to evaluate RCC cells' cell cycle distribution and apoptotic cell ratio. Our results indicated that Lv‐MEF2A significantly increased the apoptotic cell ratio compared to the vector group (Figure 6A), which suggested that MEF2A influences RCC progression by modulating RCC cell apoptosis. Additionally, our cell cycle analysis revealed a marked increase in the G1 phase cell population after Lv‐MEF2A (Figure 6B), indicating that MEF2A affects RCC progression by disturbing the G1/S transition of cell cycle. Furthermore, Bax, Bcl‐2 and Caspase‐3 are crucial proteins involved in cell apoptosis. In this study, we performed western blot to assess the expression of these proteins, and our results demonstrated that Bax and Caspase‐3 were upregulated, whereas Bcl‐2 was downregulated in the Lv‐MEF2A group compared to the vector group (Figure 6C). Therefore, we conclude that MEF2A inhibits RCC progression by arresting G1/S transition of cell cycle and promoting cell apoptosis.

### 3.5 MEF2A overexpression inhibits RCC progression by disturbing Wnt pathway

**FIGURE 7:**
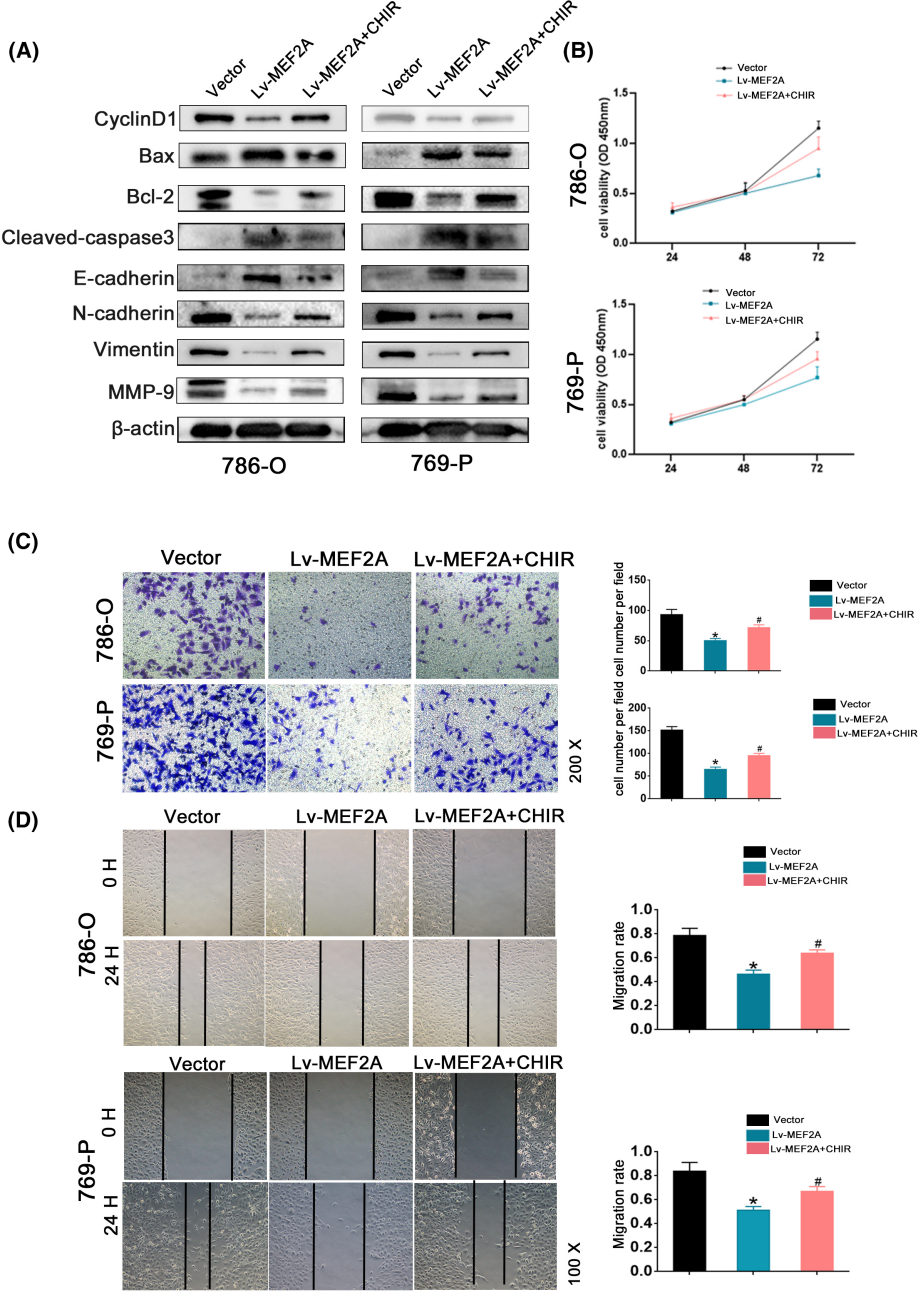
MEF2A affected renal cell carcinoma (RCC) progression after using the Wnt activator. (A) Western blot assay verified apoptosis‐associated protein expression, EMT‐associated protein expression and MMP‐9 expression after using Wnt‐ activator. (B) CCK‐8 proliferation assay after using Wnt activator. (C) Transwell assay after using Wnt activator. (D) Wound healing assay after using Wnt activator.

Through GSEA analysis (Figure 6D), we hypothesized that MEF2A influences RCC progression by affecting the Wnt pathway. To investigate this conjecture, we performed western blot assays to detect the protein expression of key players in the Wnt signalling pathway. The results revealed stable downregulation of Survivin, β‐catenin, CyclinD1 and c‐myc upon MEF2A overexpression (Figure 6E). We then used a Wnt activator (Laduviglusib, CHIR‐99021) to activate Wnt signalling. Through western blot assay, we discovered that cyclin D1, N‐cadherin, Vimentin, MMP9 and Bcl‐2 obtain expression upregulation after using CHIR‐99021; Bax, Caspase‐3 and E‐cadherin acquired expression downregulation after using CHIR‐99021 (Figure 7A). Then, we performed a CCK‐8 assay, which showed that compared with the Lv‐MEF2A group, RCC cells had increased cell proliferation ability in the Lv‐MEF2A + CHIR group (Figure 7A). Wound healing (Figure 7B) and Transwell assays (Figure 7C) provided similar results. These findings suggest that CHIR‐99021 partially restores RCC cell proliferation and invasion abilities. Therefore, we conclude that MEF2A overexpression inhibits RCC progression by suppressing the Wnt/β‐catenin signalling pathway, arresting the G1/S transition of the cell cycle and promoting RCC cell apoptosis.

### 3.6 MEF2A inhibits RCC progression in vivo

**FIGURE 8:**
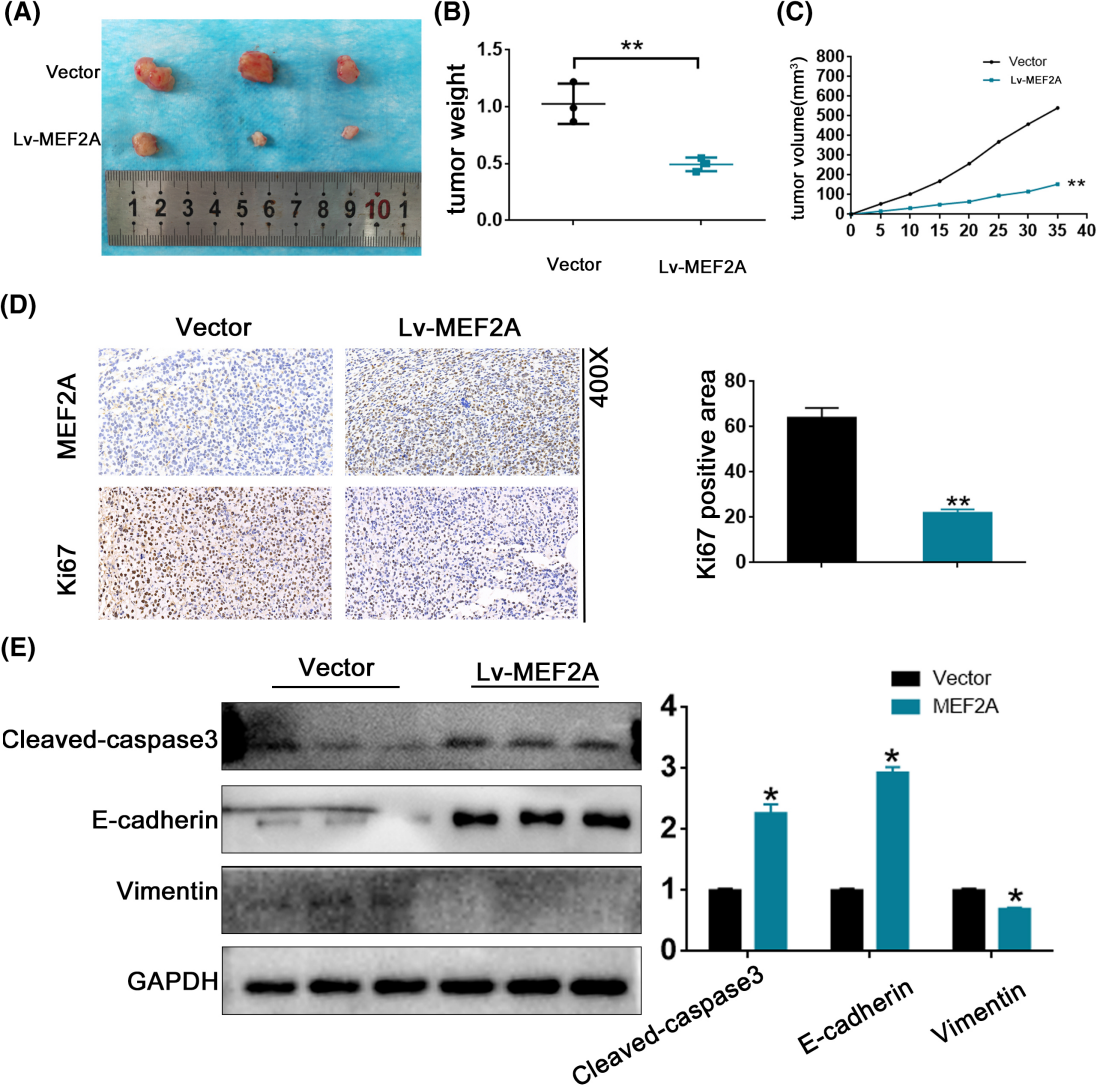
MEF2A inhibits renal cell carcinoma (RCC) progression in vivo. (A) The xenograft assay revealed tumour size is smaller in the Lv‐MEF2A group than in the Vector group. (B) The xenograft assay revealed tumour weight is lower in the Lv‐MEF2A group than in the Vector group. (C) The xenograft assay revealed tumour volume is lower in the Lv‐MEF2A group than in the Vector group. (D) Ki67 protein expression is highly expressed in Lv‐MEF2A. (E) Western blot verified protein expression of Vimentin, E‐cadherin and Caspase 3 in a xenograft assay.

To observe the impact of MEF2A on RCC progression, we injected RCC cells into mice to assess the correlation between MEF2A overexpression and cancer growth in vivo. In the xenograft assay, the Lv‐MEF2A group exhibited reduced tumour size (Figure 8A), weight (Figure 8B) and volume (Figure 8C) compared to the vector group, indicating that MEF2A can inhibit tumour growth in RCC. Additionally, we used IHC assay to test Ki67 protein expression in the Lv‐MEF2A and vector groups, which revealed that Ki67 protein expression was lower expression in the Lv‐MEF2A group than the Vector group (Figure 8D). Finally, western blot analysis was conducted to validate the expression levels of Vimentin, E‐cadherin and Caspase‐3 (Figure 8E), which aligned with the findings from the preceding cell line experiments.

We first selected MEF2A as our target in this study through bioinformatic analysis. Through a series of basic experiments, we observed that MEF2A expression is higher in normal kidney tissue than in RCC tumour tissue. We speculated that the ubiquitination led to MEF2A expression downregulation. We also found that MEF2A overexpression inhibits RCC cell proliferation, migration, invasion and EMT and that MEF2A inhibits RCC progression by disrupting the Wnt signalling pathway. These conclusions were validated in both in vitro and in vivo experiments. Therefore, we conclude that MEF2A overexpression suppresses RCC progression by inhibiting the Wnt pathway.

## 4 DISCUSSION

Renal cell carcinoma (RCC) is the main type of tumour in the urological system, accounting for 2%–3% of adult malignant cancers.^21^ RCC is the main subtype of RCC and accounts for 80% of cases.^22^ Although most RCC patients are diagnosed early, partly, patients still occur metastases after diagnosis treatment.^23^ Although targeted treatments, such as rapamycin inhibitors, are considered potential therapies for RCC patients, their effects on increasing survival time remain unclear.^24^ Therefore, this study aimed to explore a novel biomarker to improve overall survival (OS) and prognosis for RCC patients.

Brusatol, a particularly bioactive composition of Brucea javanica, has been shown to possess an antitumor function in multiple cancers.^3^ In addition, previous studies have demonstrated that brusatol inhibits RCC progression by targeting the PTEN/PI3K/AKT signalling pathway,^9^ which motivated further investigation of the potential effects of brusatol. Bioinformatics was widely applied to identify key genes.^25^ In this study, 37 brusatol‐associated genes were predicted using bioinformatics and further analysed using the CytoHubba plug‐in of Cytoscape software. The five most important genes obtained were ALB (Albumin), AR (Androgen Receptor), CCNT2 (Cyclin T2), MEF2A (Myocyte Enhancer Factor 2A) and RHOQ (Ras Homolog Family Member Q). Finally, through survival filter analysis and molecular binding energy calculation, MEF2A was selected as our objective.

MEF2A is a member of the MEF2 (myocyte enhancer factor 2) family, which plays essential roles in cell differentiation and development.^26^, ^27^, ^28^ Aberrant MEF2 expression has been linked to tumour development, including HCC (Hepatocellular Carcinoma)^29^ and CRC (Colorectal Cancer).^30^ Recently, MEF2A was reported in partly tumour progression. In CRC, MEF2A promotes tumour progression by upregulating ZEB2 (Zinc Finger E‐Box Binding Homeobox 2) and CTNNB1 (Catenin Beta 1).^31^ In breast cancer (BC), MEF2A induced tumour metastasis by affecting MMP10 expression.^32^ In gastric cancer (GC), MEF2A promotes cancer cell proliferation and metastasis by p38MAPK phosphorylation.^33^ Through survival analysis, it was found that MEF2A overexpression was closely associated with longer OS, and MEF2A also could be considered an independent prognosis factor for RCC patients. Thus, we speculated that MEF2A plays an essential role in RCC progression.

Ubiquitination is often studied in tumour progression research as a biological process that can cause protein expression downregulation. Previous studies show that BECN1 ubiquitination is closely associated with autophagy, which results in tumour progression.^34^ The Guangwei Z et al. survey found that TRAF6 ubiquitination of K63‐linked chains inhibits tumour progression.^35^ In our study, MEF2A could be considered tumour suppressor genes (TSG) inhibiting RCC progression. The ubiquitin–proteasome system (UPS) could accelerate tumour suppressor protein degradation, leading to tumour progression.^36^ Thus, our study found that MEF2A downregulation is closely associated with ubiquitination. Inhibiting MEF2A ubiquitination degradation inhibits RCC progression. Our previous study found that brusatol inhibits RCC progression by the PTEN/PI3K/AKT pathway.^9^ This study found that brusatol inhabits MEF2A ubiquitination in RCC cell lines. Thus, we concluded that MEF2A ubiquitination led to the low expression of MEF2A, and brusatol could protect MEF2A from ubiquitination degradation.

Most malignant tumours are characterized by their ability to proliferation, invasion and metastasis. Our study evaluated malignant features using a series of assays. PCNA is crucial for tumour cell DNA replication and is a marker for tumour proliferation.^20^ We found that overexpression of MEF2A led to decreased cell proliferation ability and proliferation‐associated biomarkers such as colony formation ability and PCNA infiltration intensity. Western blot assay was utilized to detect the expression of EMT‐associated markers and proliferation‐associated markers, including E‐caderin, N‐caderin, Vimentin and MMP9. Epithelial‐mesenchymal transition (EMT) is a dynamic cellular process through which epithelial cells undergo phenotypic changes, acquiring mesenchymal characteristics. Various states of EMT have been linked to enhanced proliferation, invasion and metastasis across multiple tumour types.^37^ Moreover, EMT was regarded as a hallmark of cancer stem cells and may provide insight into therapy tolerance. Therefore, targeting EMT‐associated molecules represented a potential therapy method.^38^ Our study found that the MEF2A overexpression led to the downregulation of E‐caderin and Vimentin and the upregulation of N‐caderin, indicating that RCC cells lost EMT features. Therefore, MEF2A overexpression could be a potential therapeutic direction for treating RCC patients.

The uncontrolled proliferation of cancer cells is typically associated with compensatory suppression of apoptosis and cell cycle transition.^39^ Through flow cytometry, we found that overexpression of MEF2A increased the cell number in the G1 phase and the cell apoptosis rate. Bax, Bcl‐2 and Caspase‐3 were essential proteins for apoptosis, while CyclinD1 is crucial for the cell cycle's G1 to S phase transition.^40^, ^41^, ^42^ Our western blot assay revealed that apoptosis‐associated proteins and cyclin D1 were consistent with the flow cytometry results. Therefore, we concluded that MEF2A affects RCC progression by influencing G1/S transition of cell cycle and cell apoptosis.

Additionally, our GSEA analysis revealed that MEF2A might inhibit RCC progression by the Wnt signalling pathway, which is crucial in tumour progression and influences the tumour microenvironment.^43^ Previous studies have shown that Wnt also affects tumour immune surveillance, presenting a potential novel therapy for cancer immunotherapy.^10^, ^44^ The Wnt pathway is involved in multiple tumour progressions, such as breast cancer,^45^ colorectal cancer (CRC),^46^ pancreatic cancer^47^ and others. Activating the Wnt pathway promotes tumour progression in hepatocellular carcinoma (HCC).^48^ The transition from monocyte to M2‐polarized macrophages activates the Wnt/β‐catenin signalling pathway, and co‐culture of M2‐polarized macrophages and tumour cells has a significant antitumor effect.^49^ Metastasis was the main reason for death in CRC. FUBP1 promotes CRC metastasis by activating the Wnt pathway.^50^ In ovarian cancer, Wnt2B inhibited tumour progression by downregulating the Wnt/β‐catenin pathway.^51^ PROX1 accelerated BC development by triggering the Wnt pathway.^12^ CCT5 promotes tumour progression in gastric cancer by inducing EMT and activating the Wnt pathway.^52^ In our study, overexpression of MEF2A led to decreased Wnt‐associated proteins expression, including β‐catenin and survival. Therefore, we conclude that MEF2A inhibits RCC progression by suppressing the Wnt pathway.

Our study found that MEF2A ubiquitination led to the low expression of MEF2A, and brusatol could protect MEF2A from ubiquitination degradation. MEF2A overexpression inhibits RCC progression by inhibiting the Wnt/β‐catenin pathway, arresting G1/S1 transition of cell cycle, and inducing RCC cell apoptosis.

## 5 CONCLUSION

In our study, we have determined that MEF2A exerts inhibitory effects on RCC progression by inducing cell cycle arrest at the G1/S phase, facilitating apoptosis of RCC cells and suppressing the Wnt/β‐catenin signalling pathway.

## AUTHOR CONTRIBUTIONS

**Tao Wang:** Conceptualization (equal); data curation (equal); investigation (equal); methodology (equal); project administration (equal); supervision (equal); writing – original draft (equal); writing – review and editing (equal). **Yu Zhou:** Conceptualization (equal); investigation (equal); supervision (equal); writing – original draft (equal); writing – review and editing (equal). **Hui Bao:** Data curation (equal); formal analysis (equal); investigation (equal). **Bo Liu:** Supervision (equal); validation (equal); visualization (equal). **Min Wang:** Supervision (equal); validation (equal); visualization (equal). **Lei Wang:** Project administration (equal); supervision (equal); validation (equal). **Tiejun Pan:** Funding acquisition (equal); investigation (equal); project administration (equal); supervision (equal); validation (equal); visualization (equal).

## FUNDING INFORMATION

The General Hospital of the Centrol Theater Command funded this study (NO: ZZYCZ202120).

## CONFLICT OF INTEREST STATEMENT

The authors declare no competing interests regarding this work.

## CONSENT FOR PUBLICATION

All patients signed consent for publication.

## Supporting information


Figure S1.

Figure S2.

## Data Availability

The data presented in this study are available and were acquired by the corresponding author.
