# Supplementary figures and images for: Brusatol enhances MEF2A expression to inhibit RCC progression through the Wnt signalling pathway in renal cell carcinoma
Source: J Cell Mol Med. 2023 Oct 20;27(23):3897–910. doi: 10.1111/jcmm.17972 (PMC10718142; doi:10.1111/jcmm.17972)

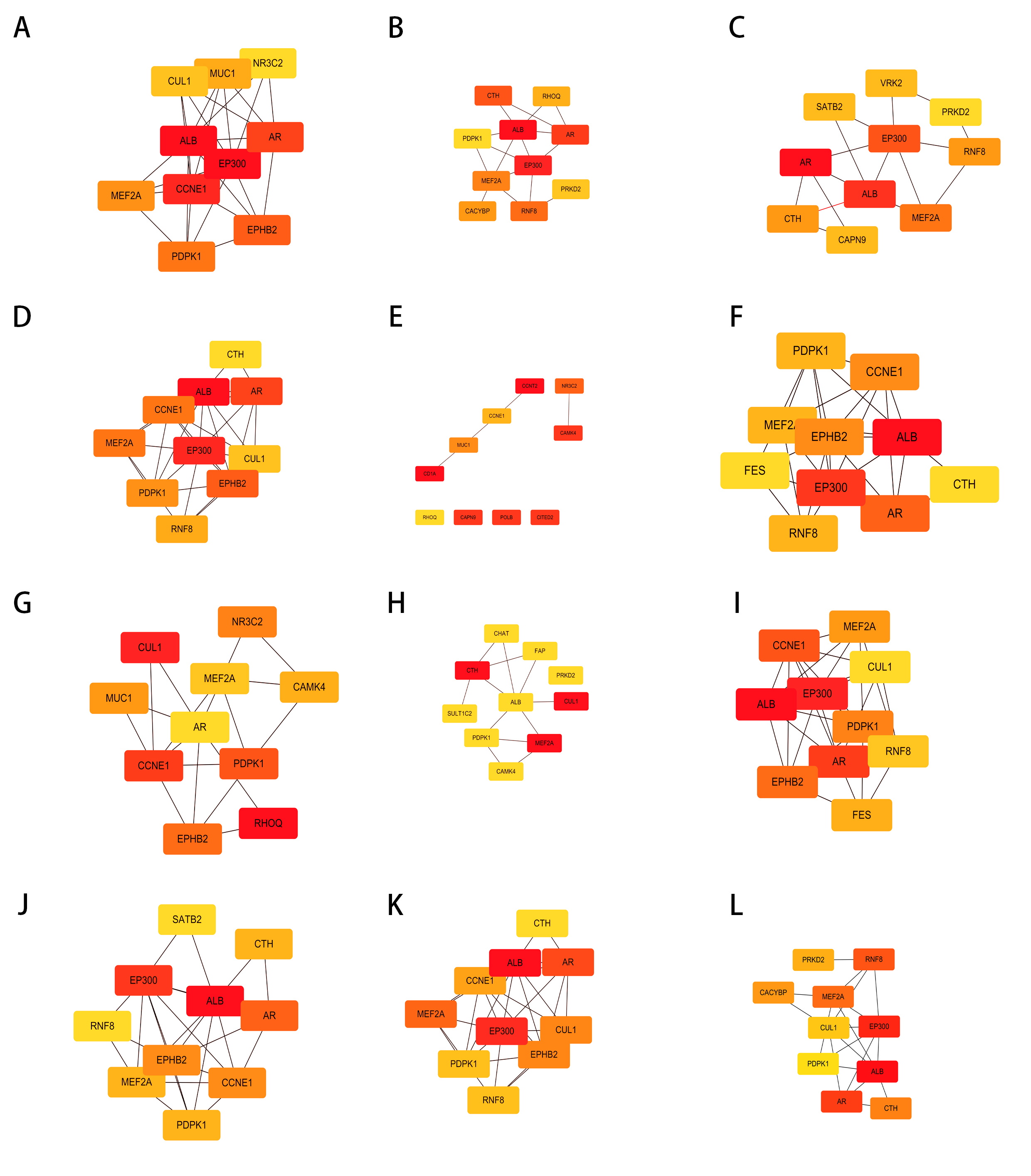

Supplement: Supplementary file 1 — Figure S1. [file JCMM-27-3897-s001.jpg]

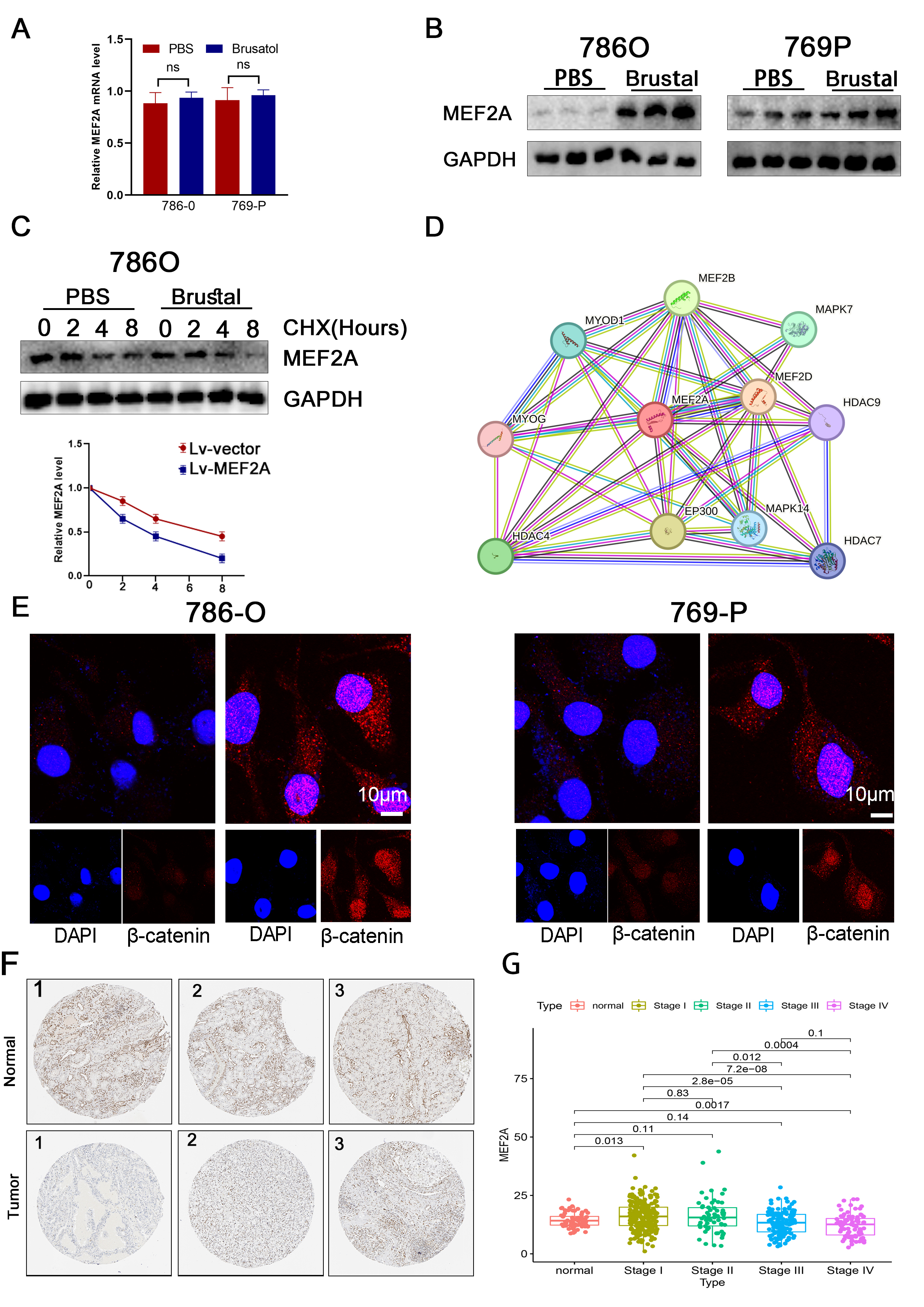

Supplement: Supplementary file 2 — Figure S2. [file JCMM-27-3897-s002.jpg]
